# Anti-GIP antibodies and future diabetes related risk: an eight-year prospective cohort study

**DOI:** 10.3389/fendo.2026.1811527

**Published:** 2026-04-29

**Authors:** Takahide Hashimoto, Naoki Ohtake, Tokiko Okamoto, Bo-Shi Zhang, Yoich Yoshida, Hirotaka Takizawa, Masaya Yamaga, Tomohiko Yoshida, Amika Kajiyama, Takeshi Yagihashi, Rikako Furukawa, Takaki Hiwasa, Minoru Takemoto

**Affiliations:** 1 Department of Diabetes, Metabolism and Endocrinology, School of Medicine, International University of Health and Welfare, Narita, Chiba, Japan; 2 International University of Health and Welfare, Atami Hospital, Atami, Shizuoka, Japan; 3 Department of Neurological Surgery, Graduate School of Medicine, Chiba University, Chu-ku, Chiba, Japan; 4 Port Square Kashiwado Clinic, Kashiwado Memorial Foundation, Chuo-ku, Chiba, Japan; 5 International University of Health and Welfare, Narita hospital, Narita, Chiba, Japan

**Keywords:** anti-GIP antibody, BMI, diabetes, immunometabolism, incretin

## Abstract

**Background:**

Autoantibodies against metabolic regulators have been implicated in metabolic disorders; however, the clinical relevance of incretin-related autoantibodies in the development of hyperglycemia remains unclear. We investigated whether autoantibodies against glucose-dependent insulinotropic polypeptide (GIP) and glucagon-like peptide-1 (GLP-1) are associated with future deterioration of glycemic status in a prospective cohort.

**Methods:**

We analyzed 218 participants who underwent health checkups and were followed for a mean of 8.1 years. Individuals with diabetes or baseline HbA1c ≥ 6.5% were excluded. Baseline serum anti-GIP and anti-GLP-1 antibody (Ab) levels were measured using AlphaLISA. Incident diabetes-range glycemia was defined as fasting plasma glucose ≥ 126 mg/dL or HbA1c ≥ 6.5% without clinical confirmation, to avoid overestimation of incident diabetes in this cohort-based setting. Predictive performance was evaluated using receiver operating characteristic (ROC) analyses. To avoid overadjustment, HbA1c was excluded from the primary prediction models.

**Results:**

During follow-up, 21 participants developed diabetes-range glycemia. Baseline anti-GIP Ab levels were significantly higher in individuals who developed diabetes-range glycemia, whereas anti-GLP-1 Ab levels showed no association with risk. Anti-GIP Ab levels alone demonstrated modest but significant discriminative ability (AUC = 0.656). Although body mass index was a strong predictor (AUC = 0.799), adding anti-GIP Ab levels modestly improved model performance (AUC = 0.819).

**Conclusions:**

Elevated baseline anti-GIP Ab levels were associated with the future development of diabetes-range glycemia and provided complementary predictive information beyond conventional metabolic risk factors. However, given the modest incremental improvement beyond BMI and the limited number of incident cases, the predictive contribution of anti-GIP antibody levels should be interpreted as exploratory and hypothesis-generating and requires validation in larger independent cohorts. These findings suggest that GIP-related immune responses may represent a distinct immunometabolic component involved in early glycemic deterioration.

## 1 Introduction

The global prevalence of type 2 diabetes mellitus (T2DM) continues to increase at an alarming rate, posing a major public health and socioeconomic challenge worldwide (1). T2DM is a progressive metabolic disorder characterized by impaired insulin secretion and insulin resistance, ultimately leading to chronic hyperglycemia and associated complications (2). Early identification of individuals at high risk of developing diabetes is essential, as timely interventions may delay or prevent disease onset and related complications. However, despite advances in diagnostic tools and preventive strategies, accurately predicting future glycemic deterioration remains challenging, representing a significant clinical concern. Incretin hormones, such as glucose-dependent insulinotropic polypeptide (GIP) and glucagon-like peptide-1 (GLP-1), play central roles in glucose homeostasis by enhancing insulin secretion in a glucose-dependent manner (3). Beyond glucose metabolism, GLP-1 signaling exerts pleiotropic effects, including cardiovascular protection, renal and neuroprotective actions, regulation of appetite and lipid metabolism, and modulation of inflammation and immune responses (4, 5). In contrast, the extra-pancreatic roles of GIP signaling remain less well characterized. Furthermore, autoantibodies, traditionally studied in autoimmune diseases, are increasingly being recognized in metabolic disorders, malignancies, and chronic inflammatory conditions. Advances in proteomic and immunoassay technologies have enabled comprehensive profiling of autoantibodies, several of which are now considered potential biomarkers for disease risk, progression, and treatment response (6). We previously reported that autoantibodies against metabolic enzymes, such as phosphoenolpyruvate carboxykinase (PCK 1) (7), proprotein convertase subtilisin/kexin type 9 (PCSK9) (8) and GIP (9), were elevated in patients with diabetes and were associated with overall survival. Based on these findings, we inferred that autoantibodies may play a role in metabolic dysregulation. Therefore, we aimed to examine the association between incretin antibodies and metabolic parameters and to evaluate the potential clinical utility of incretin antibody measurements in predicting the development of hyperglycemia beyond established metabolic risk factors.

## 2 Materials and methods

### 2.1 Collection of serum samples

This study was conducted in accordance with the ethical principles of the Declaration of Helsinki. The study protocol was approved by the Ethics Committees of the International University of Health and Welfare (Approval No. 24-Nr-005) and Chiba University (Approval Nos. 2017-251, 2018-320, and 2020-1129). All participants provided written informed consent. Serum samples were collected at the Port-Square Kashiwado Clinic and stored at -80 °C until further analysis. A total of 218 participants (96 male and 122 female) who provided written informed consent and underwent health checkups between April and October 2013 were randomly selected.

### 2.2 Preparation and purification of incretin proteins

Full-length GIP and GLP-1 cDNAs were cloned into the prokaryotic expression plasmid pGEX-4T-1. ECOS^TM^ competent *Escherichia coli* BL-21 cells (Nippon Gene; Tokyo, Japan) were transformed with pGEX-4T-1, pGEX-4T-1-GIP, and pGEX-4T-1-GLP-1 and cultured for 3 h in 200 mL of Luria broth containing 0.1 mM isopropyl β-D-thiogalactopyranoside (Wako Pure Chemicals, Osaka, Japan). The cells were then lysed by sonication in BugBuster Protein Extraction Reagent (Merck Millipore, Darmstadt, Germany). GST, GST-GIP, and GST-GLP-1 proteins were purified as previously described (10).

### 2.3 Measurement of serum antibody levels

Serum levels of antibodies against GIP and GLP-1 were measured using an amplified luminescent proximity homogeneous assay-linked immunosorbent assay (AlphaLISA) performed in 384-well white opaque microtiter plates (OptiPlate™, Revvity, Waltham, MA, USA) as previously described (11, 12). The assay employed glutathione-conjugated donor beads that bind GST-tagged fusion antigens and anti-human IgG–conjugated acceptor beads that detect antigen-bound immunoglobulins. Binding of serum antibodies to GST–GIP or GST–GLP-1 fusion proteins brings donor and acceptor beads into proximity, generating luminescence upon excitation at 680 nm and emission detection at 607–623 nm.

Each well contained 2.5 μL of 1:100 diluted serum and 2.5 μL of antigen solution (GST, GST–GIP, or GST–GLP-1; 10 μg/mL) prepared in AlphaLISA immunoassay buffer. After incubation at room temperature for 6–8 hours, donor and acceptor beads were added (2.5 μL each at 40 μg/mL), followed by incubation in the dark. Luminescence signals (Alpha photon counts) were measured using an EnSpire Alpha microplate reader (Revvity) according to the manufacturer’s instructions. Antigen-specific signals were calculated by subtracting background luminescence obtained using GST alone from signals obtained with GST-fusion proteins.

To minimize technical variability, all serum samples were stored at −80 °C prior to measurement and analyzed using a standardized protocol consistent with our previously validated studies of metabolic autoantibodies. Assay procedures followed identical buffer composition, antigen preparation, bead concentrations, and signal acquisition settings across measurements. In addition, relative antibody signal intensities were interpreted primarily as comparative values within the cohort rather than absolute quantitative antibody concentrations.

### 2.4 Clinical definitions

The diabetes-range group was defined as fasting glucose ≥126 mg/dL or HbA1c ≥6.5%. Hypertension was defined as blood pressure ≥130/80 mmHg according to the Japanese Society of Hypertension guidelines. Dyslipidemia was diagnosed based on the criteria of the Japan Atherosclerosis Society.

### 2.5 Statistical analysis

Continuous variables were expressed as mean ± standard deviation, and categorical variables as frequencies and percentages. Correlations between anti-GIP Ab levels, anti-GLP-1 Ab levels, and clinical characteristics were evaluated using Spearman’s rank correlation analysis. Comparisons between the diabetes-range and non-diabetes-range groups were performed using the Mann–Whitney U test for continuous variables and the chi-square test for categorical variables.

For predictive analyses, receiver operating characteristic (ROC) curves were constructed to evaluate the ability of baseline body mass index (BMI) and anti-GIP Ab levels to discriminate between the diabetes-range and non-diabetes-range groups. The area under the ROC curve (AUC) was calculated, and optimal cutoff values were determined using the Youden index, with the corresponding sensitivity and specificity reported. To further assess predictive performance, a simple additive model combining normalized BMI and anti-GIP Ab levels was evaluated. Missing values were handled using case-wise deletion (complete case analysis). Differences in AUC between models were assessed using nonparametric bootstrap resampling, as the DeLong test was not applicable to this modeling framework.

A two-sided p < 0.05 was considered statistically significant. Statistical analyses were performed using Python (version 3.11; Python Software Foundation, Wilmington, DE, USA) with the Scikit-learn, Pandas, and SciPy libraries.

## 3 Results

### 3.1 Cross-sectional associations between incretin antibodies and baseline clinical parameters

Participants with a prior diagnosis of T2DM, a history of anti-diabetic treatment, or baseline HbA1c ≥ 6.5% were excluded. The baseline clinical characteristics of the 218 participants (96 male and 122 female) who underwent health checkups in 2013 are summarized in **Table 1**.

**Table 1:** Baseline clinical characteristics of study participants in 2013.

Value	Mean ± SD
Age (year)	51.5 ± 8
Height (cm)	163.7 ± 8.7
Weight (kg)	62.5 ± 13.8
BMI (kg/m^2^)	23.2 ± 3.8
Total Protein (g/dL)	7.2 ± 0.5
Albumin (g/dL)	4.4 ± 0.3
AST(IU/L)	22.6 ± 8.1
ALT (IU/L)	22.2 ± 14.2
γ-GT (IU/L)	34 ± 35.6
HbA1c (%)	5.6 ± 0.3
LDL-cholesterol (mg/dL)	124.9 ± 31.1
Total cholesterol (mg/dL)	206.3 ± 32
Triglycerol (mg/dL)	101.1 ± 89
HDL-cholesterol (mg/dL)	68.5 ± 20
BUN (mg/dL)	14.1 ± 4
CRE (mg/dL)	0.8 ± 0.2
eGFR (mL/min/1.73 m^2^)	77.3 ± 12.3
Uric acid (mg/dL)	5.2 ± 1.3
CRP (mg/mL)	0.2 ± 0.1
Anti-GIP Ab (a.c.)	6069 ± 4269.4
Anti-GLP-1 Ab (a.c.)	1243.8 ± 2413.9

Data are presented as mean ± standard deviation or median (interquartile range), as appropriate.

The anti-GLP-1 and anti-GIP antibody (Ab) levels were determined using the AlphaLISA assay, and the values are expressed as alpha counts (a.c.).

BMI, body mass index; AST, aspartate aminotransferase; ALT, alanine aminotransferase; γ-GT: γ-glutamyltransferase, HbA1c, hemoglobin A1c; LDL, low-density lipoprotein; HDL, high-density lipoprotein; CRE, creatinine; BUN, blood urea nitrogen; eGFR: estimated glomerular filtration rate, CRP, C-reactive protein; GLP-1: glucagon like 1, GIP: glucose-dependent insulinotropic polypeptide.

At baseline, anti-GIP Ab levels showed weak but significant correlation with several metabolic parameters, including age and C-reactive protein, as shown in **Table 2**. Anti-GLP-1 Ab levels showed positive correlations with weight and lipid parameters, as shown in **Table 3**.

**Table 2:** Correlations between anti-GIP antibody levels and clinical characteristics were evaluated using Spearman’s rank correlation analysis.

Variable	N	Spearman_rho	Spearman_p
Gender	218	-0.081	0.2346
Age	**218**	**0.15**	**0.0266**
Height	218	0.055	0.4209
Weight	218	0.087	0.2
BMI	218	0.092	0.175
Total Protein	214	-0.103	0.134
Albumin	211	0.006	0.9346
AST	218	-0.05	0.4657
ALT	218	-0.02	0.7701
γ-GTP	218	0.025	0.7093
BUN	185	0.015	0.8359
CRE	215	0.062	0.3672
eGFR	215	-0.091	0.1826
Uric acid	217	0.039	0.5689
LDL-cholesterol	218	0.045	0.5058
Total cholesterol	218	-0.004	0.9516
Triglycerol	218	0.104	0.1256
HDL-cholesterol	218	-0.118	0.0812
HbA1c	210	0.116	0.0922
CRP	**207**	**0.147**	**0.0348**

Statistical significance was defined as a p value < 0.0**5. **Statistically significant values are shown in bold.

**Table 3:** Correlations between anti-GLP-1 antibody levels and clinical characteristics were evaluated using Spearman’s rank correlation analysis.

Variable	N	Spearman_rho	Spearman_p
Gender	218	-0.1	0.14
Age	218	0.088	0.194
Height	218	0.117	0.0846
Weight	**218**	**0.133**	**0.0492**
BMI	218	0.114	0.0921
Total Protein	214	-0.05	0.4695
Albumin	211	-0.041	0.5524
AST	218	0.024	0.728
ALT	218	0.074	0.2738
γ-GTP	218	0.069	0.3106
BUN	185	0.018	0.8109
CRE	215	0.107	0.1192
eGFR	215	-0.086	0.2093
Uric acid	217	0.092	0.1769
LDL-cholesterol	**218**	**0.158**	**0.0192**
Total cholesterol	218	0.095	0.1617
Triglycerol	**218**	**0.175**	**0.0098**
HDL-cholesterol	**218**	**-0.195**	**0.0039**
HbA1c	210	0.107	0.122
CRP	207	0.03	0.6684

Statistical significance was defined as a p value < 0.05. Statistically significant values are shown in bold.

Overall, baseline levels of both incretin antibodies exhibited only weak correlations with metabolic parameters and showed no association with HbA1c, indicating limited cross-sectional relationships between incretin Ab levels and conventional clinical markers among healthy individuals.

### 3.2 Associations between incretin antibodies and clinical parameters after 8 years of follow-up (longitudinal analysis)

After a mean follow-up period of 8.1 ± 2.9 years, anti-GIP Ab levels showed a negative correlation with HDL-cholesterol (HDL-C) (ρ = −0.155, p = 0.02). In contrast, anti-GLP-1 antibodies showed a positive correlation with triglycerol (ρ = 0.145, p = 0.033), consistent with previous observations, and a negative correlation with HDL-C (ρ = −0.235, p = 0.0005).

### 3.3 GIP antibody levels were elevated in participants who developed diabetes-range glycemia

During the follow-up period, 21 of the 218 participants (6.0%) developed diabetes-range glycemia, defined as fasting blood glucose ≥ 126 mg/dL or HbA1c ≥ 6.5%. Comparisons of baseline clinical characteristics showed that HbA1c had the strongest association with incident diabetes-range glycemia, followed by obesity-related indices, including BMI and body weight. Notably, anti–GIP Ab levels emerged as a novel factor distinct from conventional metabolic risk markers, suggesting the possible involvement of an additional incretin-related pathway in the development of diabetes-range glycemia. In contrast, lipid abnormalities, liver enzyme elevations, systemic inflammation, and uric acid levels appeared to play supportive but secondary roles (**Table 4**).

**Table 4:** Comparison of baseline characteristics and laboratory parameters between diabetes range glycemia (DRG) (defined as fasting blood glucose (FBS) ≥ 126 mg/dL or HbA1c ≥ 6.5%) and non-diabetes range glycemia (non-DRG).

Variable	DRG_mean	DRG_SD	Non-DRG_mean	Non-DRG_SD	p-value
Anti-GIP Ab	38173.1	13464.7	30416.5	13744.1	0.019
AST	32.2	21.6	21.1	12.7	0.005
γ-GT	52.3	35.4	32.0	35.1	0.000
Weight	72.0	14.4	61.4	13.4	0.000
BMI	26.8	3.7	22.8	3.6	0.000
HbA1c	5.9	0.3	5.5	0.2	0.000
Triglycerol	136.8	86.6	97.3	88.6	0.012
HDL-cholesterol	60.7	19.7	69.3	19.8	0.022
Uric acid	6.0	1.1	5.1	1.2	0.001
CRP	0.16	0.11	0.12	0.08	0.0053

Group comparisons were performed using the Mann–Whitney U test for continuous variables and the chi-square test for categorical variables. This table presents only variables that differed significantly between the two groups (*p* < 0.05). SD: standard deviatio.n

**Figure 1:**
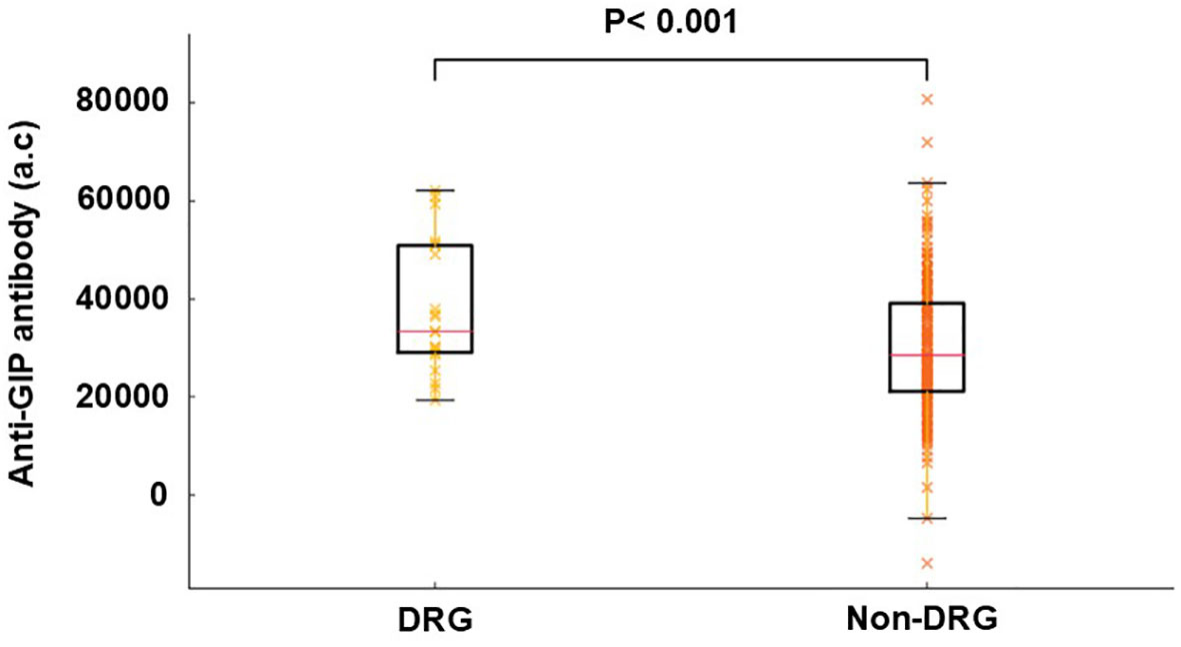
Comparison of serum anti-GIP antibody levels between diabetes range glycemia (DRG) and non-diabetes range glycemia (non-DRG) groups. Boxplots indicate the median and interquartile ranges of anti-GIP antibody levels, whereas individual data points are overlaid to illustrate the distribution within each group. Anti-GIP antibody levels were significantly higher in the DRG group compared with that in the non-DRG group (p < 0.05, Mann–Whitney U test).

Baseline anti–GIP Ab levels were significantly higher in participants who subsequently developed diabetes-range glycemia than in those who did not (38,173.1 ± 13,464.7 vs. 30,416.5 ± 13,744.1; p = 0.019) (**Figure 1**). In contrast, anti–GLP-1 Ab levels did not differ significantly between the two groups (23,698.3 ± 11,860.6 vs. 18,785.4 ± 11,423.7, p = 0.115).

### 3.4 Multivariable analysis adjusted for metabolic confounders

**Figure 2:**
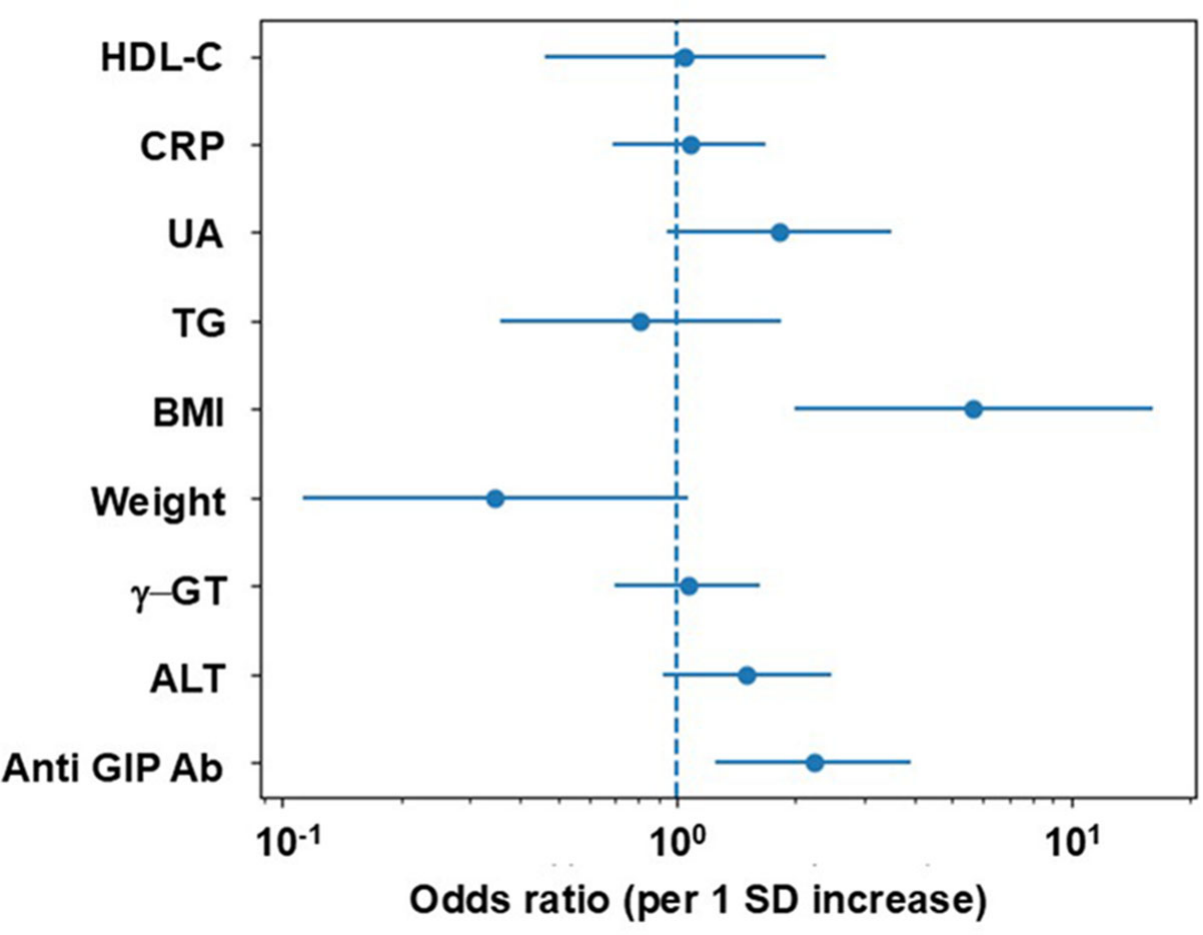
Independent association of anti GIP Ab with the development of diabetes-range glycemia in a multivariable logistic regression model. Forest plot showing standardized odds ratios (per 1 SD increase) for diabetes-range glycemia based on a multivariable logistic regression model excluding HbA1c. The dashed line represents an odds ratio of 1.0.

Because HbA1c is included in the definition of diabetes-range glycemia, it was excluded from multivariable analyses to avoid overadjustment and circular reasoning. **Figure 2** presents a forest plot of odds ratios derived from a standardized multivariable logistic regression model excluding HbA1c. After adjustment for BMI and other baseline metabolic parameters, anti-GIP Ab remained independently associated with the development of diabetes-range glycemia (odds ratio per 1 SD increase, 2.22; 95% confidence interval, 1.25–3.95; p = 0.0066).

In contrast, most conventional metabolic markers, including liver enzymes, lipid parameters, and inflammatory markers, did not show statistically significant independent associations.

In univariate analyses, anti-GIP Ab levels were significantly correlated with age and C-reactive protein (CRP). Therefore, age and CRP were included as covariates in additional multivariable models. Even after adjustment for age, CRP, BMI, and other baseline metabolic parameters, anti-GIP Ab remained independently associated with the development of diabetes-range glycemia.

### 3.5 Identification of factors associated with incident diabetes-range glycemia

**Figure 3:**
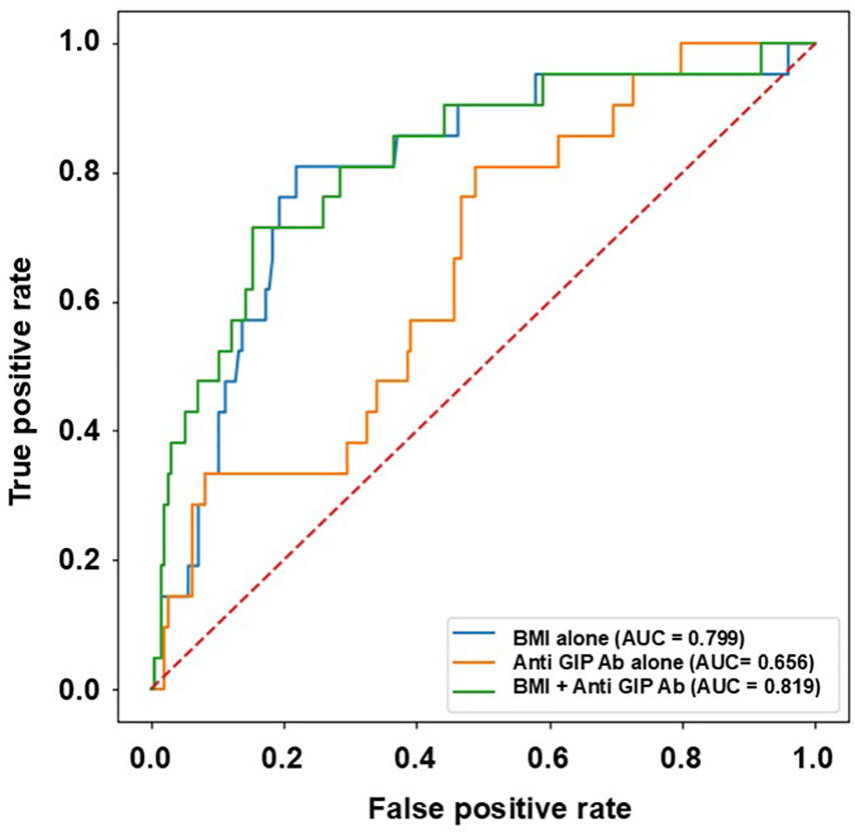
Receiver operating characteristic (ROC) curves for diabetes prediction using different biomarkers. ROC curves for the prediction of diabetes-range glycemia using body mass index (BMI) alone, anti-GIP Ab alone, and a combined model incorporating both BMI and anti-GST Ab. The area under the curve (AUC) was 0.799 for BMI alone, 0.656 for anti-GST Ab alone, and 0.819 for the combined model. The dashed diagonal line represents the reference line for no discrimination (AUC = 0.5).

Receiver operating characteristic (ROC) curve analyses were performed to evaluate the predictive performance of BMI alone, anti–GIP Ab alone, and their combination for diabetes-range glycemia. The area under the curve (AUC) was 0.799 for BMI alone and 0.656 for anti–GIP Ab alone. Notably, the combined model incorporating both BMI and anti–GIP Ab yielded a higher AUC of 0.819, indicating improved discrimination compared with either variable alone (**Figure 3**). Although the addition of anti-GIP antibody levels to BMI improved the AUC from 0.799 to 0.819, this incremental improvement was modest and should be interpreted cautiously. These findings suggest that anti-GIP antibody levels may provide complementary immunometabolic information rather than functioning as standalone predictive biomarkers.

Using the Youden index, the optimal cutoff value for anti GIP Ab–based prediction was determined. At this cutoff, sensitivity was 81.0% and specificity was 51.3%, indicating that elevated anti-GIP Ab levels may help identify individuals at increased risk of developing diabetes-range glycemia.

## 4 Discussion

To the best of our knowledge, this is the first study to evaluate the clinical significance of measuring autoantibodies against incretins in healthy participants. Correlation analyses showed weak but significant associations between anti-GIP Ab levels with age and C-reactive protein, whereas anti-GLP-1 Ab levels showed meaningful correlations with weight and lipids. Although these correlations were modest, suggesting limited clinical significance, the observed patterns imply that anti-GIP and anti-GLP-1 antibodies may exert different biological effects.

Both GIP and GIP-1 co-stimulate insulin secretion from pancreatic β-cells; however, they also exert distinct extra-pancreatic actions (13). For example, GIP primarily affects bone and adipose tissue, whereas GLP-1 acts on the heart, liver, and kidney (14, 15). Accordingly, antibodies against GIP or GLP-1 may influence clinical parameters. Because anti-GLP-1 antibodies were associated with lipid levels and body weight, they may be linked to atherosclerosis or steatotic liver disease (SLD). However, atherosclerotic or SLD outcomes were not assessed in this cohort and future studies are warranted to further investigate this hypothesis.

In the longitudinal cohort, among the 218 participants who underwent health checkups and were followed for a mean of 8.1 years, baseline anti-GIP Ab levels were significantly higher in individuals who subsequently developed diabetes-range glycemia than in those who did not, whereas anti-GLP-1 Ab levels were not associated with future hyperglycemia risk.

ROC analyses demonstrated that BMI was a strong independent predictor of diabetes-range development (AUC = 0.799). Anti-GIP Ab levels alone showed modest but significant discriminative ability (AUC = 0.656). The addition of anti-GIP Ab levels to the BMI-based model increased the AUC to 0.819. Notably, even in the absence of HbA1c, incorporation of anti-GIP Ab levels consistently improved the predictive performance of models based on obesity (BMI), supporting its potential role as a complementary biomarker reflecting a distinct pathophysiological axis. Although the improvement in AUC after adding anti-GIP antibody levels to BMI-based prediction models was modest, this finding suggests that anti-GIP antibodies may reflect an additional immunometabolic axis complementary to conventional obesity-related risk factors rather than functioning as a standalone predictive biomarker. Because BMI itself already represents a strong predictor of future diabetes-range glycemia, even a modest increase in discrimination may reflect complementary biological information rather than a large improvement in clinical prediction performance. Given the limited number of incident cases in this cohort, this incremental improvement should be interpreted as exploratory and hypothesis-generating, and requires confirmation in larger independent cohorts.

However, the mechanisms underlying the association between anti-GIP antibodies and the development of diabetes-range glycemia remain unclear. Given that GIP acts on adipocytes to regulate energy storage and lipid handling (16–18), the presence of anti-GIP antibodies may disrupt GIP signaling in adipose tissue, potentially leading to dysregulation of adipocytokine secretion and subsequent impairment of glucose metabolism, thereby contributing to the development of diabetes-range glycemia. Although the improvement in AUC after adding anti-GIP antibody levels to BMI-based prediction models was modest, this finding suggests that anti-GIP antibodies may reflect an additional immunometabolic axis complementary to conventional obesity-related risk factors rather than functioning as a standalone predictive biomarker. Given the limited number of incident cases in this cohort, this incremental improvement should be interpreted as exploratory and hypothesis-generating, and requires confirmation in larger independent cohorts.

Our findings are consistent with previous reports showing that autoantibodies against metabolic regulators, including PCK1 (7), PCSK9 (8), and GIP, are elevated in patients with diabetes and are associated with poorer overall survival. The present study extends these observations by suggesting that immune responses targeting incretin-related pathways may also contribute to metabolic risk and disease progression.

Although anti-GIP Ab levels demonstrated lower predictive power for diabetes-range glycemia than BMI, their significant elevation in individuals who developed diabetes-range glycemia supports a potential link between GIP-related immunity and metabolic deterioration. These findings indicate that anti-GIP antibodies may serve not as standalone diagnostic markers but as complementary indicators reflecting a distinct immunometabolic axis involved in the pathogenesis of diabetes.

The recent success of dual GIP/GLP-1 receptor agonists provides additional context for our findings. Tirzepatide has been shown to substantially delay progression from prediabetes to type 2 diabetes in large clinical trials, indicating that intact GIP signaling plays a protective role in metabolic regulation (19). Therefore, elevated anti-GIP antibody levels observed in our cohort may reflect impaired incretin signaling, which could predispose individuals to glycemic deterioration.

This study has several limitations. First, it was a single-center study with the number of incident diabetes-range glycemia cases in this cohort was relatively small (n = 21), which may have limited the statistical stability of multivariable analyses and increased the risk of overfitting. Therefore, the predictive contribution of anti-GIP antibody levels should be interpreted cautiously and considered exploratory and hypothesis-generating. Validation in larger independent cohorts will be necessary to confirm the robustness and generalizability of these findings. In addition, the strict definition of diabetes-range glycemia (fasting plasma glucose ≥ 126 mg/dL and HbA1c ≥ 6.5%) reduced the number of events available for analysis, precluding more extensive statistical evaluation.

Second, the observational nature of the study does not allow causal relationships to be established. Third, functional experiments were not conducted to directly assess whether anti-GIP antibodies interfere with incretin signaling or downstream metabolic pathways.

Several additional limitations should be considered when interpreting the present findings. Although anti-GIP Ab levels were measured using an AlphaLISA-based immunoassay with GST-fusion antigens and background subtraction using GST controls, additional validation experiments such as recovery assays using spiked antibodies or affinity purification approaches were not performed in this study. Therefore, further confirmation will be required to fully establish the antigen specificity of the detected signals. In addition, low-level antigen-reactive antibodies against endogenous regulatory peptides have been reported in apparently healthy individuals and may reflect physiological immunometabolic activity rather than disease-specific autoimmunity. Future studies incorporating affinity-based validation and functional characterization will be necessary to clarify the biological nature and functional role of anti-GIP Ab. Accordingly, the present findings should be interpreted as exploratory evidence of an immunometabolic association rather than definitive proof of disease-specific autoantibodies.

Despite these limitations, the present findings highlight a potential role of anti-GIP Ab in the early phase of glycemic deterioration. Although conventional metabolic factors such as BMI remain important contributors to diabetes risk, the observed association between elevated anti-GIP Ab levels and future diabetes-range glycemia suggests that GIP-related immune responses may represent an additional and distinct biological component of metabolic risk. These results support further investigation of incretin-related autoimmunity in larger prospective cohorts.

In conclusion, this study suggests that immune responses directed against GIP may be involved in the development of diabetes-range glycemia. While obesity-related factors provide a metabolic background for hyperglycemia risk, anti-GIP antibodies may capture complementary immunometabolic information not fully explained by conventional clinical markers. Larger, multicenter studies incorporating mechanistic approaches are warranted to clarify whether anti-GIP antibodies and other metabolic autoantibodies can serve as meaningful biomarkers of metabolic risk and potentially contribute to the pathogenesis of diabetes.

## Data Availability

The original contributions presented in the study are included in the article/supplementary material. Further inquiries can be directed to the corresponding author/s.
